# A *cis*-element at the *Rorc* locus regulates the development of type 3 innate lymphoid cells

**DOI:** 10.3389/fimmu.2023.1105145

**Published:** 2023-03-09

**Authors:** Dehui Chang, Hao Zhang, Jing Ge, Qi Xing, Xinyi Guo, Xiaohu Wang, Chen Dong

**Affiliations:** ^1^ Institute for Immunology and School of Medicine, Tsinghua University, Beijing, China; ^2^ Shanghai Immune Therapy Institute, Shanghai Jiao Tong University School of Medicine Affiliated Renji Hospital, Shanghai, China; ^3^ Tsinghua University-Peking University Center for Life Sciences, Tsinghua University, Beijing, China; ^4^ Research Unit of Immune Regulation and Immune Diseases of Chinese Academy of Medical Sciences, Shanghai Jiao Tong University School of Medicine-Affiliated Renji Hospital, Shanghai, China

**Keywords:** ILC3s, RORγt, CNS9, cis-element, plasticity

## Abstract

**Background:**

As an important early source of IL-17A and IL-22 in immune responses, type 3 innate lymphoid cells (ILC3s) are critically regulated by the transcription factor retinoic-acid-receptor-related orphan receptor gamma t (RORγt). Previously, we have identified a crucial role of the conserved non-coding sequence 9 (CNS9), located at +5,802 to +7,963 bp of the *Rorc* gene, in directing T helper 17 differentiation and related autoimmune disease. However, whether *cis*-acting elements regulate RORγt expression in ILC3s is unknown.

**Results:**

Here we show that CNS9 deficiency in mice not only decreases ILC3 signature gene expression and increases ILC1-gene expression features in total ILC3s, but also leads to generation of a distinct CD4^+^NKp46^+^ ILC3 population, though the overall numbers and frequencies of RORγt^+^ ILC3s are not affected. Mechanistically, CNS9 deficiency selectively decreases RORγt expression in ILC3s, which thus alters ILC3 gene expression features and promotes cell-intrinsic generation of CD4^+^NKp46^+^ ILC3 subset.

**Conclusion:**

Our study thus identifies CNS9 as an essential *cis*-regulatory element controlling the lineage stability and plasticity of ILC3s through modulating expression levels of RORγt protein.

## Introduction

Innate lymphoid cells (ILCs), important in defense against invading pathogens at the mucosal surface, contain three major subpopulations depending on their distinct gene expression patterns. Group 3 ILCs (ILC3s), similar to T helper 17 (Th17) cells, express transcription factor RORγt and secrete interleukin-17A (IL-17A) and/or IL-22 (1, 2). ILC3s play important roles in controlling extracellular bacteria and fungi, as well as tissue repair following mucosal barrier damage (3).

ILC3s are generally divided into three major subsets in mice, based on expression of chemokine receptor CCR6 and natural cytotoxicity receptor NKp46. CCR6^+^(NKp46^-^) ILC3s include lymphoid tissue inducer (LTi) and LTi-like cells, are heterogenous in CD4 expression, which are indispensable for lymphoid tissue formation (4). CCR6^+^(NKp46^-^) ILC3s have been reported to facilitate CD4^+^/CD8^+^ T cells in anti-tumor response (5). NKp46^+^(CCR6^-^) ILC3s are pathogenic in anti-CD40-induced innate colitis model, through recruiting inflammatory monocytes (6). However, the role of CCR6^-^NKp46^-^ double-negative ILC3s has not been extensively characterized. Intestinal NKp46^+^(CCR6^-^) ILC3s, developed from NKp46^-^CCR6^-^ ILC3s upon up-regulating T-bet expression, have mixed features of both ILC3s and ILC1s, with expression of NKp46, T-bet and interferon γ (IFN-γ), and can convert to ILC1-like cell or even ILC1s (7–11). ILC3s are thus highly plastic, readily in response to continuously changing microenvironments.

As the lineage-specific transcription factor of both Th17 cells and ILC3s, RORγt (encoded by *Rorc*) is essential for their development and effector functions. Conserved non-coding sequences (CNSs) are regulatory *cis*-acting elements critically controlling gene expression *via* interaction with various *trans*-acting factors. A number of CNSs have been identified at the *Rorc* locus. CNS9 and CNS6 are required for IL-6-STAT3 and TGF-β-SMAD&c-MAF signaling, respectively (12). Interestingly, deletion of CNS6 or CNS9 did not affect the development of γδT cells or ILC3s. Therefore, the *cis*-regulatory mechanisms controlling *Rorc* transcription in ILC3s remain unclear. Comparative transposase-accessible chromatin using sequencing (ATAC-seq) and RNA-seq analysis revealed many potentially active *cis*-elements in ILCs (13), but none of them have been functionally and genetically analyzed.

In this study, we discovered that CNS9 deficiency altered ILC3 features in intestine, associated with increased ILC1 but decreased ILC3 signature gene expression. Moreover, loss of CNS9 reduced RORγt expression at per cell level and causes induction of specific CD4^+^NKp46^+^ ILC3 subset that is not found in healthy WT mice. Our results thus indicate that *cis*-regulatory element CNS9 is important in maintaining the lineage stability or development of ILC3s.

## Materials and methods

### Mice

C57BL/6J and CD45.1^+^ mice were obtained from Jackson Laboratory, and were crossed to generate CD45.1^+^CD45.2^+^ mice. CNS9-deficient mice were generated on C57BL/6J background by using CRISPR-Cas9 system in our previous study (12). RORγt^GFP^ mice were generated by the laboratory of Prof. Dan R. Littman (4).

All mice were housed in specific pathogen-free (SPF) conditions and in isolated ventilated cages in the animal facility at Tsinghua University, which has been accredited by AAALAC (Association for Assessment and Accreditation of Laboratory Animal Care International). All the animal protocols used in this study has been approved by IACUC (Institutional Animal Care and Use Committee) of Tsinghua University. All mice used in this study were at 6-15 weeks, age and sex matched.

### Isolation of immune cells

Lamina propria lymphocytes (LPLs) were isolated from small intestine or large intestine. The intestine was collected by removing mesenteric lymph nodes and Peyer’s patches and cut into 2-3 cm pieces longitudinally, then digested at 37°C for 30 mins in RPMI 1640 medium containing 5 mM EDTA, 20 mM HEPES, 1 mM DTT and Penicillin/Streptomycin. The tissues were washed twice using RPMI 1640 medium containing 2mM EDTA, 20mM HEPES and Penicillin/Streptomycin. The remaining tissues were chopped into small pieces and digested in digestion buffer (RPMI 1640 medium with 20 mM HEPES, 0.5 mg/ml collagenase D, 1 mg/ml Dispase and 2 mg/ml DNase I and Penicillin/Streptomycin) at 37°C for 30 min. Then the tissues were meshed through 100 μm cell strainer for cells suspension. LPLs were obtained from the interface of 70% and 40% Percoll after centrifuging at 2500 rpm, 25°C for 30 min.

Spleen, mesenteric and inguinal lymph nodes were taken out, ground and passed through 100 μm cell strainer (spleen needed additional lysing to remove red blood cells).

### *C. rodentium* infection model

Mice were fasted for 7-8 hours and then orally gavaged with 2*10^9^ CFU *C. rodentium*/mouse. The body weight and fecal bacterial load were monitored. If indicated, the mice were sacrificed and intestinal LPLs were analyzed 8 days post-infection.

### *Ex vivo* stimulation

To measure IFN-γ expression, ILC3s were stimulated at 37°C for 4 hours with 25 ng/ml IL-12, 50 ng/ml IL-15 and 5 ng/ml IL-23 in the presence of GolgiStop™. While for measuring IL-22 and IL-17A, cells were stimulated at 37°C for 4 hours with 5 ng/ml IL-23 in the presence of GolgiStop™. As specifically indicated, IL-22 production in **Figure S2B** was measured with IL-12 and IL-15 stimulation.

### Generation of RORγt overexpression chimeric mice

On day 0, bone marrow cells were isolated from CNS9-deficient mice, depleted red blood cells and then cultured in 6-well plates (4×10^6^ cell for each well) in RPMI 1640 medium (20 ng/mL IL-3, 50 ng/mL IL-6 and 50 ng/mL SCF with Penicillin/Streptomycin) at 37°C in a CO_2_ incubator. On day 2, polybrene was added into the cell mixture at a final concentration of 8 μg/mL. Bone marrow cells were infected with RVKM control or RVKM-RORγt retrovirus, centrifuged at 1800 rpm for 2 hours at 35°C. This infection was repeated on day 3. On day 4, the virus infected GFP^+^ bone marrow cells were sorted, and then intravenously injected into lethally irradiated (2 rounds of 5Gy irradiation, an interval of 2 hours) CD45.1^+^ recipient mice at 2 x 10^6^/mouse. Seven weeks later, the recipient mice were sacrificed and analyzed.

### Antibiotic treatment

CNS9-deficient mice were treated with antibiotics (ampicillin: 1 g/L, vancomycin: 500 mg/L, neomycin: 1 mg/mL and metronidazole: 1 mg/mL) starting from 3 to 6 weeks old. The antibiotics were supplied in drinking water and changed every two days to keep fresh.

### ScRNA-seq library construction and data processing

ILC3 (CD45^mid^ CD3^-^ CD90.2^high^) cells were sorted from small intestine LPLs using flow cytometry. Single-cell RNA libraries were generated using 10X Genomics chromium according to the manufacturer’s instructions. The quality of libraries was determined by Agilent 2100 bioanalyzer and sequenced with NovaSeq 6000 PE150(Illumina).

To generate single cell feature counts, the clean data of each sample were mapped to mouse reference genome (mm10) by the command “cell ranger count” within cell ranger toolkit (version 6.0.1) provided by 10X genomics. For each sample, cells as outliers in feature count matrix (proportion of mitochondrial genes > 10% and ncount_feature < 300 or ncount_feature > 4700) were removed from further analysis. To remove impure cells, we first filtered the cells by *Ptprc* ^+^ *Cd3e* ^-^ *Thy1* ^+^ according to sorting strategy, and then exclude non-ILC3 based on signature gene expression, including T cells (*Cd3g, Cd3e*), B cells (Cd79a, IgIv1, Jchain), macrophages (*Cd68, Apoe, C1qb, Csf1r*), endothelial cells (*Fabp4, Id3, Lyve1* (14)), ILC1/NK (*Ccl5, Klrd1, Klrc2, Eomes*) and ILC2s (*Klrg1, Il4, Il5, Il13*) (15). Finally, we obtained 6860 cells and 4517 cells from WT and CNS9KO samples respectively.

### Clustering of single cell data matrix

The Seurat R package (version 4.0.3) was conducted for further analysis of feature count matrix. For each sample, about 2,000 genes with the highest variance based on a variance stabilizing transformation data were selected to compute a PCA dimensionality reduction. Integration of two samples was conducted by RunHarmony() function in Seurat R package. The 30 principal components (PCs) of the integrated Seurat object were used for further nonlinear dimensional reduction analysis. The integrated data were clustered using Seurat’s FindClusters() function (resolution parameters was set to 0.8), then visualized by UMAP. The different expression of the selected marker genes in each cluster were visualized by DotPlot() function.

### RNA velocity analysis

For each sample, a.loom file with counts divided in spliced/unspliced/ambiguous was generated by velocyto.py toolkit (version 0.17). Then, the data were pre-processed by scVelo python toolkit (version 0.2.4). Genes that are detected in less than 20 counts were filtered out and 2000 genes with the highest variability were selected. After normalization and logarithmization, the first and second order moments (means and uncentered variances) were computed among nearest neighbors in PCA space under default parameters. Then, the RNA velocity was estimated by scVelo’s scv.tl.velocity() function, and visualized by scv.pl.velocity_embedding_stream() function.

### Generation of bone marrow chimeric mice

Bone marrow cells from C57BL/6J, CNS9-deficient and CD45.1^+^CD45.2^+^ mice were isolated and depleted red blood cells. C57BL/6J or CNS9-deficient bone marrow cells were mixed with the ones from CD45.1^+^CD45.2^+^ mice at 1:1 ratio, and 4×10^6^ mixed bone marrow cells were injected intravenously into lethally irradiated (2 rounds of 5Gy irradiation, an interval of 2 hours) CD45.1^+^ recipient mice. The reconstituted mice were then sacrificed and analyzed 7 weeks later.

### Flow cytometry analysis

The antibodies and corresponding dilutions listed below were used for staining: CD45.2 (104, 109824, BioLegend; 104, 56-0454, eBioscience; 1:400), CD45.1 (A20, 558701, BD BioSciences; 1:400), CD3 (17A2, 48-0032-82, eBioscience; 1:400), CD3e (145-2C11, 45-0031-82, eBioscience; 1:200), CD90.2(53-2.1, 25-0902-82/17-0902-83, eBioscience; 1:400), CD4(RM4-5, 562314/557956, BD BioSciences; RM4-5, 25-0042-81, eBioscience; 1:400), CCR6 (29-2L17, 129819, BioLegend; 1:200), NKp46 (29A1.4, 137606, BioLegend; 29A1.4, 25-3351-82, eBioscience; 1:400), RORγt (Q31-378, 562607/562682, BD BioSciences; 1:400), T-bet (4B10, 644814, BioLegend: 1:400), IL-17a (eBio17B7, 48-7177-82, eBioscience; 1:200), IL-22(IL22JOP, 17-7222-82, eBioscience; 1:200), IFN-γ (XMG1.2, 557998, BD BioSciences; 1:200).

### Quantification and statistical analysis

Data were analyzed using Graphpad Prism 6 or Graphpad Prism 8 and preformed as mean ± SD. Statistical significance was calculated using unpaired Student’s t test and shown as (*p < 0.05; **p < 0.01; ***p < 0.001, ****p < 0.0001).

## Results

### CNS9 deficiency alters intestinal ILC3 subsets

**Figure 1:**
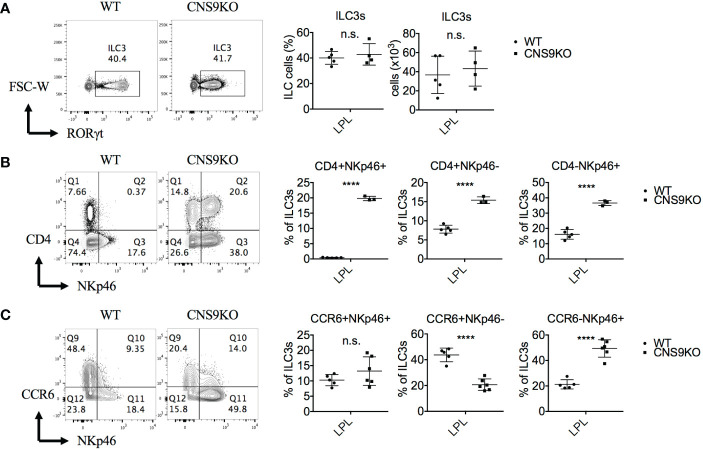
CNS9 deficiency alters the composition of ILC3s. ILC3s were isolated from the small intestine lamina propria lymphocytes (LPLs) of age- and sex-matched WT and CNS9-deficient (CNS9KO in figure) mice under steady state, directly stained with indicated surface markers and RORγt, and then analyzed by flow cytometry. **(A)** Left: intracellular staining of RORγt (pre-gated on Live CD45^+^ CD3^-^ CD90^+^); right: statistic of the staining data and cell number of ILC3s. Staining (left) and statistic (right) of the surface marker CD4 and NKp46 **(B)** or CD4 and CCR6 **(C)** on ILC3s (pre-gated on ILC3: Live CD45^+^ CD3^-^ CD90^+^ RORγt^+^). The data shown are a representative of two to three independent experiments and presented as mean ± SD. Also see **Figure S1**. Statistical significance was calculated using unpaired Student’s t test and shown as ****p < 0.0001 or n.s. (non-significant) p > 0.05.

We have previously identified CNS9, located at +5,802 to +7,963 bp of the *Rorc* gene, whose deficiency in mice caused a profound defect in *Rorc* expression and RORγt-directed IL-17A expression in Th17 cells (12). In contrast, CNS9 deficiency had little effect on development of total RORγt^+^ ILC3s (gated on CD45^+^CD3^-^CD90^+^) in small intestine under steady state, as examinbed in both percentages and numbers (**Figure 1A**). In addition, under *C. rodentium* infection state, CNS9-deficient mice showed comparable degrees of weight loss and bacterial loads at the early stage of infection to control WT mice (**Figures S1A, B**). These data suggest that the general development and function of ILC3s were largely unaffected by CNS9 deficiency.

However, a careful examination revealed that CNS9 deficiency significantly increased expression of both CD4 and NKp46 in ILC3s in the LPLs of small intestine under steady state, resulting in a clear population of CD4^+^NKp46^+^ double positive cells (**Figure 1B**). As introduced previously, CD4 only expressed by part of CCR6^+^ ILC3s but not NKp46^+^ ILC3s, suggesting the CD4^+^NKp46^+^ ILC3 subset in CNS9-deficient mice is a distinct population compared with WT mice. In addition, the expression of CCR6 was found decreased in CNS9-deficient ILC3s (**Figure 1C**), suggesting the composition of ILC3 subsets was significantly altered in CNS9-deficient mice, despite their overall function may not be affected. Consistently, CD4^+^NKp46^+^ ILC3s were also present in the lamina propria of both small and large intestines in CNS9-deficient mice 8 days after *C. rodentium* infection (**Figures S1C, D**), as well as in other lymphoid tissues including mesenteric lymph nodes (mLN), inguinal lymph nodes (iLN) and spleen under steady status (**Figure S1E**), though less prominent than in gut-associated mucosal tissues.

**Figure 2:**
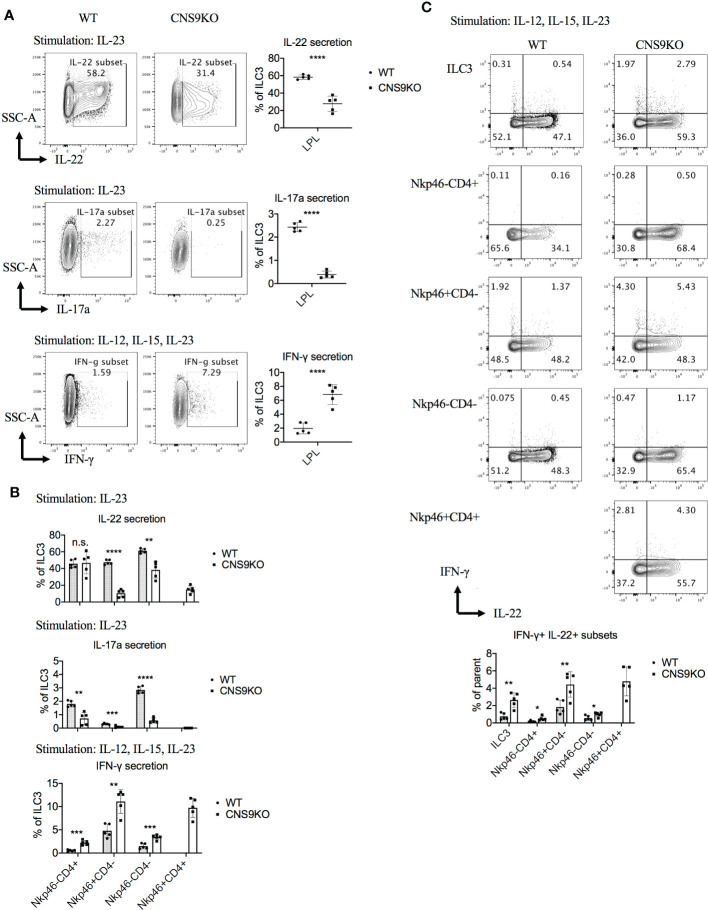
CNS9 deficiency modulates the cytokine production of ILC3s. ILC3s were isolated from the small intestine lamina propria lymphocytes (LPLs) of age- and sex-matched WT and CNS9-deficient mice under steady state. Stimulated *ex vivo* for 4 hours before staining with surface markers, RORγt and cytokines, and then analyzed by flow cytometry. **(A)** Left: intracellular staining of IL-22, IL-17A and IFN-γ single positive cells in total ILC3s; right: statistic of the staining data. **(B)** Statistic analysis of IL-22, IL-17A and IFN-γ in different ILC3 subsets. **(C)** Up: intracellular staining of IL-22 and IFN-γ; down: statistic analysis of IL-22^+^IFN-γ^+^ double positive cells in total ILC3s and different subsets. The data shown are a representative of two to three independent experiments and presented as mean ± SD. Also see **Figure S2**. Statistical significance wascalculated using unpaired Student’s t test and shown as *p < 0.05; **p < 0.01; ***p < 0.001, ****p < 0.0001 or n.s. (non-significant) p > 0.05.

At cytokine levels, CNS9-deficient ILC3s secreted 50% to 80% less IL-22 and IL-17A, but about 3-fold increase of IFN-γ, compared with WT ILC3s (**Figure 2A**). The CD4^+^NKp46^+^ ILC3s showed similar cytokine producing abilities to CD4^-^NKp46^+^ ILC3s in CNS9-deficient mice, with reduced IL-22 and increased IFN-γ but no IL-17A expression, compared with other subsets (**Figure 2B**). Moreover, associated with increased IFN-γ secretion, an obvious population of IFN-γ and IL-22 double positive cells was detected in NKp46^+^ ILC3s in CNS9-deficient mice (**Figure 2C**). These results indicate that CNS9-deficient ILC3s gained increased ILC1-related cytokine secretion potential at the expense of reduced ILC3 effector function.

Notably, there was no defect in IL-22 production in CNS9-deficient ILC3s under stimulation with IL-12, IL-15 and IL-23 condition (**Figure S2A**), which was different from cells stimulated with IL-23 alone (**Figure 2A**). Interestingly, IL-12 and IL-15 treatment could also induce IL-22 production in ILC3s, in the absence of IL-23 (**Figure S2B**), suggesting distinct mechanisms involved in IL-23- and IL-12/15-stimulated conditions.

We further measured IL-22 production in ILC3s after *C.rodentium* infection, but with only a slightly decreased trend in CNS9-deficient ILC3s compared with WT cells (**Figure S2C**), indicating a compensatory mechanism or context-dependent regulation of IL-22 production in ILC3s by CNS9 as revealed in **Figures 2A**, **S2A**. In line with this, CNS9-deficient mice showed comparable weight loss and fecal bacterial loads after *C. rodentium* infection (**Figures S1A, B**).

### CNS9 deficiency promotes cell-intrinsic induction of CD4^+^NKp46^+^ ILC3s

**Figure 3:**
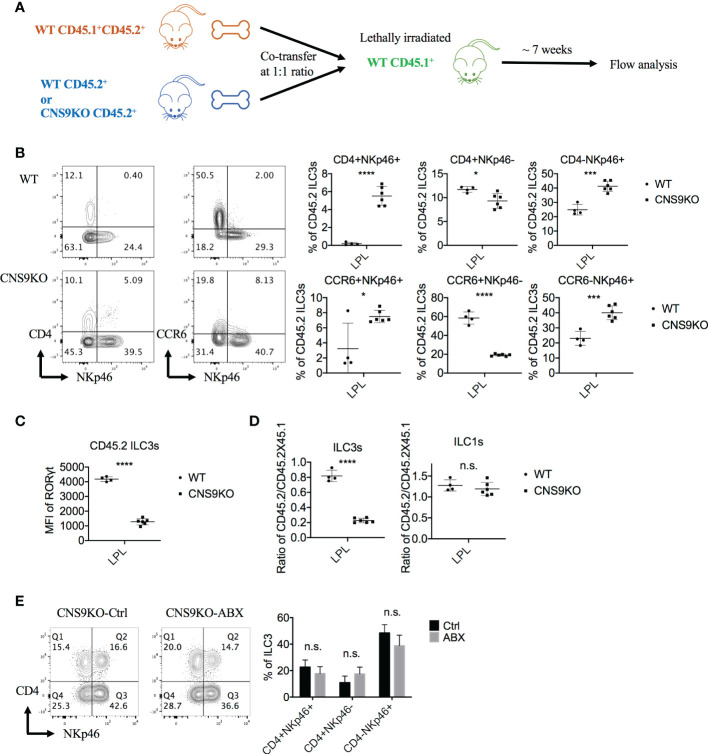
CNS9 deficiency promotes the generation of CD4^+^NKp46^+^ ILC3 subset *via* a cell-intrinsic manner. **(A)** Experiment protocol: The CD45.1^+^CD45.2^+^ WT BM cells and CD45.2^+^ CNS9-deifient BM cells were transferred to irradiated CD45.1^+^ mice at a 1:1 ratio. Seven weeks later, the recipient mice were sacificed and the small intestine LPLs were collected and analyzed by flow cytometry. **(B)** The ILC3s were gated as Live CD3^-^ CD90^+^ RORγt^+^ CD45.2^+^ cells. Left: surface staining of CD4, NKp46, CCR6 of ILC3s; right: statistic of the staining data. **(C)** MFI of RORγt in CD45.2^+^ WT and CNS9-deficient ILC3s. **(D)** The ratios of CD45.2^+^ WT/CNS9-deficient *versus* CD45.1^+^CD45.2^+^ WT ILC3s or ILC1s. The data shown are a representative of two independent experiments and presented as mean ± SD. **(E)** CNS9-deficient mice were treated with ABX or water (as control) continuously started from 3 weeks after wean. The LPLs of small intestine were isolated and analyzed at the age of 6 weeks old. Left: surface staining of CD4 and NKp46 (gated on ILC3s); right: statistic of the staining data. The flow data shown are a representative of two independent experiments and the statistical analysis was performed by combining two experiments and presented as mean ± SD (n=4 for both Ctrl and ABX group). Statistical significance was calculated using unpaired Student’s t test and shown as *p < 0.05; ***p < 0.001, ****p < 0.0001 or n.s. (non-significant) p > 0.05.

Since RORγt and its isoform RORγ can be expressed by a number of cell subsets, including Th17 cells, γδT cells, NKT cells and even non-immune cells, CNS9 deficiency may affect ILC3 development through an indirect manner. To test this possibility, mixed bone marrow chimeric mice were generated by reconstituting lethally irradiated CD45.1^+^ mice with WT CD45.1^+^CD45.2^+^ bone marrow cells and CD45.2^+^ WT or CNS9-deficient bone marrow cells at a 1:1 ratio (**Figure 3A**). 7 weeks after reconstitution, a distinct group of CD4^+^NKp46^+^ ILC3s was developed from CNS9-deficient but not WT bone marrow cells (**Figure 3B**), similar to CNS9 germline knockout mice. In addition, CNS9 deficiency led to significantly increased NKp46^+^ but decreased CCR6^+^ ILC3 populations, similar to unmanipulated mice at steady state, but the overall frequencies of CD4-expressing ILC3s were largely not affected in the chimeric animals (**Figure 3B** and **Figure 1B**). Similar to those from CNS9-deficient mice, ILC3s developed from CNS9-deficient bone marrow cells in the chimeric mice showed reduced RORγt expression level compared with those from WT bone marrow cells (**Figure 3C**). These results thus demonstrate that CNS9 functions in a cell-intrinsic manner in constraining the development of CD4^+^NKp46^+^ ILC3 subset. However, unlike CNS9-deficient mice, CNS9-deficient bone marrow cells showed a severe defect in development towards ILC3s but not ILC1s, compared with WT bone marrow cells, suggesting their apparent disadvantage in ILC3 development under competitive microenvironments (**Figure 3D**).

ILCs, abundant at mucosal barriers, are regulated by microbiota in development, maintenance and function (15, 16). To examine whether the development of CD4^+^NKp46^+^ ILC3s in CNS9-deficient mice was regulated by intestinal microbiota, we treated 3-week old mice with antibiotics (ampicillin, vancomycin, neomycin and metronidazole) supplied in drinking water for 3 weeks to remove gut microbiota. Compared with water-treated control group, antibiotic treatment did not affect the population of CD4^+^NKp46^+^ ILC3s in CNS9-deficient mice (**Figure 3E**), suggesting their generation in CNS9-deficient mice were independent of gut-associated microbiota.

### CNS9-deficient ILC3s exhibit increased ILC1 gene expression features

**Figure 4:**
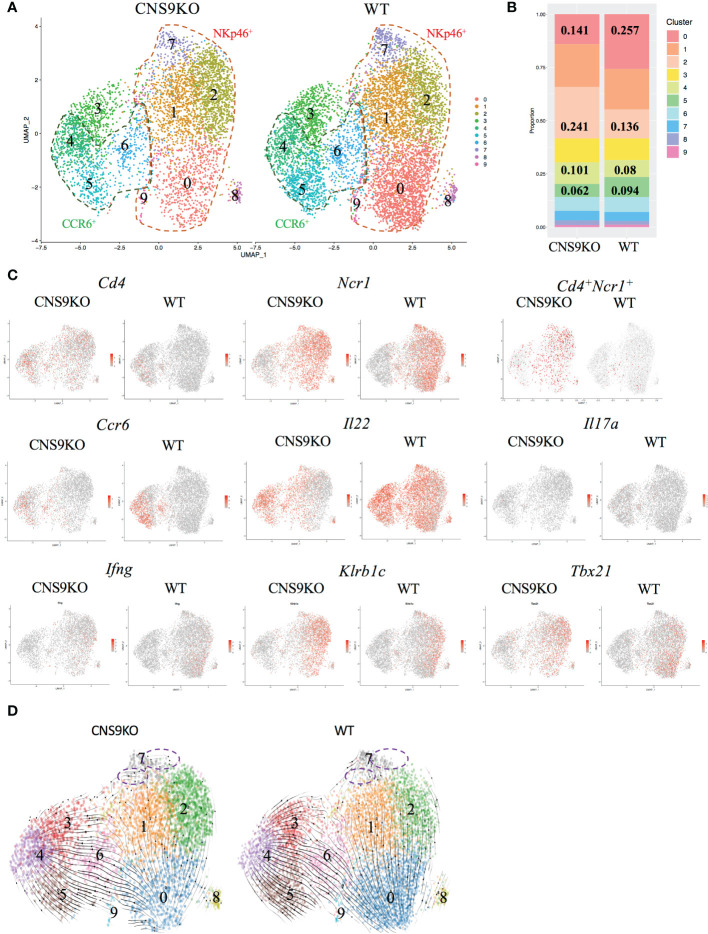
Single cell RNA-seq analysis of ILC3s isolated from CNS9-deficient mice and WT mice. The ILC3s (CD3^-^CD90.2^high^CD45^mid^) were sorted from small intestine LPLs of WT and CNS9-deficient mice, and single-cell RNA libraries were constructed using 10x Genomics. **(A)** UMAP plots of CNS9-deficient and WT ILC3s. **(B)** Statistic quantification of individual clusters between WT and CNS9-deficient ILC3s. **(C)** Selected gene expression of UMAP plots. **(D)** RNA velocity analysis among clusters. Purple dotted circle indicating RNA velocity direction from Cluster 7 to other clusters. Also see **Figure S3**.

To further understand the function of CNS9, total ILC3s (gated on CD45^mid^ CD3^-^ CD90.2^high^) were sorted from the LPLs of small intestine in WT and CNS9-deficient mice and analyzed by single-cell RNA sequencing. In total, 10 distinct clusters were identified after removing contaminated cells (**Figures 4A, B**). A previous study from Ido Amit lab (15) has defined a set of uniquely expressed genes in ILC1, ILC2 and ILC3, based on the RNA-seq data of intestinal ILC1s (CD45^+^ Lin^-^ CD127^+^ RORγt^–^ NKp46^+^), ILC2s (CD45^+^ Lin^-^ CD127^+^ RORγt^–^ KLRG-1^+^) and ILC3s (CD45^+^ Lin^-^ CD127^+^ RORγt^+^) in mice, which were used as different ILC signature genes in this study. Clusters 0, 1, 2, 7 and 9 were characterized as NKp46^+^ ILC3s due to high expression of *Ncr1* (encoding NKp46) and *Tbx21* (encoding T-bet). Cluster 2 also expressed ILC1-related signature genes, such as *Ccl5* and *Xcl1*, and may represent ILC1-like ILC3s. Clusters 4, 5 and 6 had the highest *Ccr6* expression, thus representing CCR6^+^ ILC3s (**Figures 4A**, **S3A**). Consistently, cluster 5 also had the highest expression of *Ltb* (encoding lymphotoxin beta), a gene involved in lymphoid tissue development. Cluster 4 had high H2-Aa expression, indicating a MHCII-dependent antigen presentation potential. Cluster 6 expressed high levels of *Il17a, Il17f* and *Il22*, representing mature or effector ILC3s. Cluster 3 expressed neither CCR6 nor NKp46 and was characterized as IL-22 producing CCR6^-^NKp46^-^ ILC3s. Clusters 0 and 5 expressed high amount of *Zfp36*, a negative regulator for TNF-α and GM-CSF (17). Cluster 7 highly expressed *Tnfrsf9* (encoding CD137 or 4-1BB). Cluster 8 highly expressed *Ifit1*, a gene involved in induction of type I interferons. Cluster 9 was identified as actively proliferating ILC3s due to high expression of *Mki67* and *Stmn1* (**Figure S3A**).

In the UMAP plots analysis, CNS9-deficient ILC3s contained clearly more single *Cd4* or *Ncr1* expressing and co-expressing cell populations, with reduced *Ccr6*-expressing population (**Figure 4C**). For effector cytokines secreted by ILC3s, CNS9 deficiency resulted in decreased *Il22* and *Il17a* but increased *Ifng* transcription, consistent with the results obtained by flow cytometry in CNS9-deficient mice (**Figures 4C**, **2A**). In addition, CNS9-deficient ILC3s contained cells with increased expression of *Klrb1c* (encoding NK1.1), *Tbx21* (encoding T-bet), *Ccl5* and *Xcl1*, at both population and per cell levels (**Figures 4C**, **S3B**), indicating CNS9-deficient ILC3s acquired an ILC1-gene expression feature, when compared with WT ILC3s. Gene set variation analysis (GSVA) further confirmed our hypothesis that CNS9 deficiency increased expression of ILC1 signature genes but decreased expression of ILC3 signature genes (15) (**Figure S3C**).

We further analyzed the relationships of cell clusters by RNA velocity. Increased frequency of cluster 2 and decreased frequency of cluster 0 (**Figure 4B**), along with the velocity direction from cluster 0 to cluster 1 and cluster 2 (**Figure 4D**), indicated a strong conversion potential from cluster 0 to cluster 2 in CNS9-deficient NKp46^+^ ILC3s. Similarly, decreased cluster 5 and increased cluster 4 represented a more flexible change from cluster 5 to cluster 4 in CCR6^+^ ILC3 subset after CNS9 deficiency (**Figures 4B, D**). Moreover, cluster 7, ending with all velocity arrows, represented as a fully differentiated cluster in WT ILC3s, but still had a transition potential towards other clusters in CNS9-deficient ILC3s (**Figure 4D**). Taken together, these results suggest that genetic ablation of CNS9 in ILC3s increased their plasticity and conversion potentials towards ILC1-like cells.

### Decreased cellular RORγt expression causes induction of CD4^+^NKp46^+^ ILC3 subset

**Figure 5:**
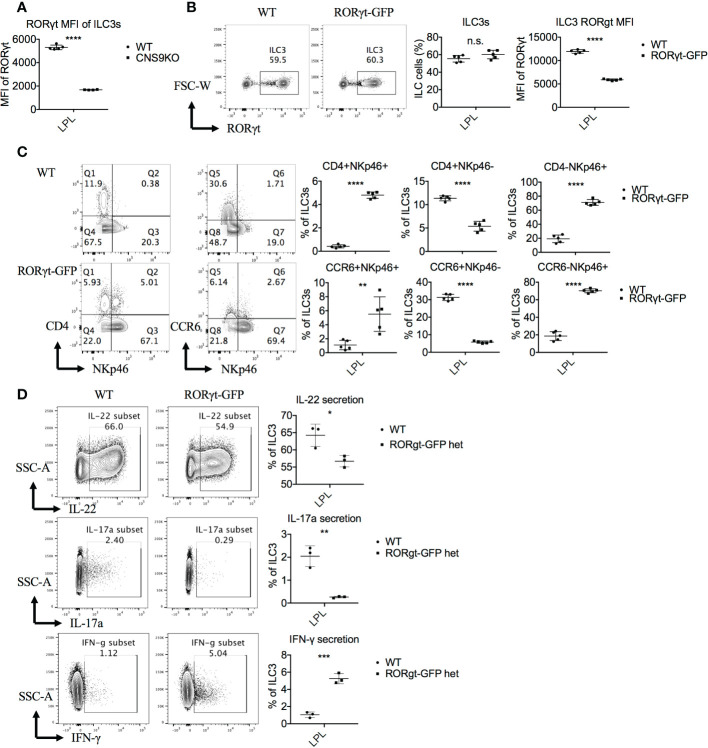
RORγt expression levels regulate the generation of the CD4^+^NKp46^+^ ILC3 subset. ILC3s were isolated from small intestine LPLs of age- and sex-matched WT and CNS9-deficient mice **(A)** or RORγt^GFP^ mice **(B–D)** under steady state. Stimulated *ex vivo* for 4 hours before staining with surface markers, RORγt and cytokines, and then analyzed by flow cytometry. **(A)** MFI of RORγt in WT and CNS9-deficient ILC3s. **(B)** Left: intracellular staining of RORγt (pre-gated on Live CD45^+^ CD3^-^ CD90^+^); right: statistic of the staining data and MFI of RORγt. **(C)** Left: surface staining of CD4, NKp46 and CCR6 of ILC3s; right: statistic of the staining data. **(D)** Left: intracellular staining of IL-22, IL-17A and IFN-γ after stimulation (pre-gated on ILC3s: Live CD45^+^ CD3^-^ CD90^+^ RORγt^+^); right: statistic of the staining data. The data shown are a representative of three independent experiments and presented as mean ± SD. Also see **Figures S4** and **S5**. Statistical significance was calculated using unpaired Student’s t test and shown as *p < 0.05; **p < 0.01; ***p < 0.001, ****p < 0.0001 or n.s. (non-significant) p > 0.05.

Our scRNA-seq data showed that *Rorc* transcription was decreased in CNS9-deficient ILC3s compared with WT ILC3s, also with a trend following velocity directions (cluster 0 -> cluster 1&2 and cluster 5 -> cluster 4) (**Figure S4A**), suggesting an internal link between *Rorc* expression levels with cluster variability. CNS9-deficient ILC3s showed increased *Cd4* transcription (**Figure S4B**). Of note, cluster 4 had lower *Rorc* but higher *Cd4* expression, compared with cluster 5, in both WT and CNS9-deficient ILC3s (**Figures S4A, B**), suggesting a potential negative regulation of *Cd4* by RORγt in ILC3s. Consistently, CNS9-deficient ILC3s had reduced RORγt protein expression at per cell levels, as examined by flow cytometry, compared with WT ILC3s (**Figure 5A**). Specifically, the subset of CD4^+^NKp46^+^ cells in CNS9-deficient ILC3s showed the lowest RORγt expression (**Figure S4C**), indicating CNS9-directed RORγt expression plays a critical role in restricting induction of CD4^+^NKp46^+^ ILC3s, likely in a dosage-dependent manner.

To test the above possibility, we examined ILC3s in the heterozygous RORγt^GFP^ mice, in which the *Gfp* gene was inserted into the first exon of *Rorc* and thus disrupted ~50% of RORγt expression at per cell levels (**Figure 5B**). Similar to CNS9-deficient mice, RORγt^GFP^ mice contained a distinct CD4^+^NKp46^+^ ILC3 subset (**Figure 5C**), and showed increased NKp46 and IFN-γ expression in total ILC3s, but a reciprocal decrease of CCR6, IL-22 and IL-17A, though CD4 expression was normal, compared with control WT mice (**Figures 5C, D**).

**Figure 6:**
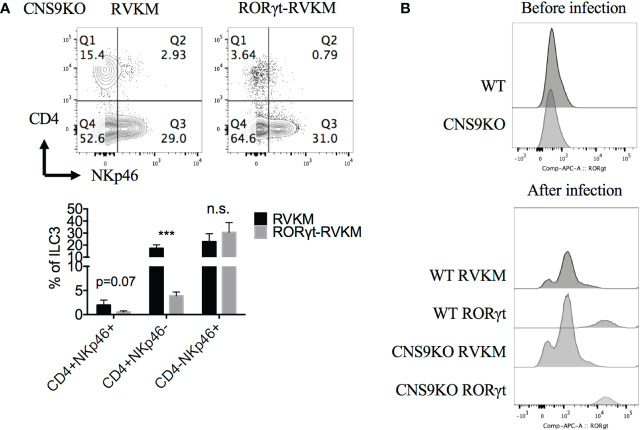
Reduced RORγt expression after CNS9 deficiency are the cause of altered ILC3 subsets. RV-GFP control or RORγt-expressing plasmids were transduced into CNS9-deficient BM cells by retro-virus infection. The infected GFP^+^ cells were sorted and transferred into irradiated CD45.1^+^ mice introveneously. ILC3s from small intestine LPLs were gated as Live CD45^+^ CD3^-^ CD90^+^ RORγt^+^ cells. **(A)** Up: surface staining of CD4, NKp46 in ILC3s; down: statistic of the staining data (n=4 for RVKM group and n=3 for RORγt -RVKM group). **(B)** RORγt protein staining in WT or CNS9-deficient BM cells before (up) and after (down) retro-virus infection (after retro-virus infection, BM cells were pre-gated on live GFP^+^ cells). The flow data shown are a representative of two independent experiments and the statistical analysis was performed by combining two experiments and presented as mean ± SD. Also see **Figure S6**. Statistical significance was calculated using unpaired Student’s t test and shown as ***p < 0.001 or n.s. (non-significant) p > 0.05.

To further confirm the dosage effect of RORγt protein, *Rorc* was retrovirally overexpressed in CNS9-deficient bone marrow cells (CD45.2^+^) *in vitro*, then transferred into irradiated CD45.1^+^ mice. 7 weeks after reconstitution, the mice were sacrificed and analyzed. Overall, *Rorc* overexpression reduced ~70% of CD4^+^NKp46^+^ ILC3s compared with control empty vector (**Figure 6A**), suggesting that decreased RORγt expression indeed contributes to the generation of CD4^+^NKp46^+^ ILC3s in CNS9-deficient mice. For unknown reasons, control retro-virus infection also promoted overall cellular RORγt expression (**Figure 6B**) and decreased the frequency of CD4^+^NKp46^+^ ILC3s compared with mice under steady state.

It has been reported previously that T-bet is critical for generation of NKp46^+^ ILC3s *via* inducing IFN-γ and NKp46 expression (8–10). We further confirmed our scRNA-seq data with flow cytometry, and found that CNS9-deficient ILC3s, including ILC3 subsets, had increased T-bet protein expression compared with WT cells (**Figure S5**). Thus, this result suggests the decreased RORγt expression directly promoted T-bet expression, and further resulted in upregulation of IFN-γ and NKp46.

CNS9-deficient ILC3s showed gain of ILC1-like gene expression feature (**Figures 4C** and **S3B, C**), increased T-bet (**Figure S5**) and IFN-γ expression (**Figure 2A**), suggesting that RORγt may regulate the balance between ILC3s and ILC1s, *via* its dosage expression. However, the percentages and cell numbers of ILC1s were only slightly increased, whereas the ratio of ILC3s to ILC1s was not significantly altered in CNS9-deficient mice, compared with WT control mice (**Figure S6**). These suggest that CNS9-directed *Rorc* transcription is not involved in transition between ILC3s and ILC1s, and CD4^+^NKp46^+^ ILC3s represent a transient state fixed by reduced but not abolished RORγt expression in CNS9-deficient mice.

## Discussion

*Cis*-regulatory mechanisms have been extensively investigated in different T helper cell subsets, such as Th1, Th2 and Th17 cells, but remain largely unclear in their corresponding counterparts of innate immune cells. In previous studies, we showed that CNS9 deficiency abolished RORγt expression and Th17 cell differentiation *in vitro* and *in vivo*. However, this study reveals that CNS9 deficiency did not affect the generation of RORγt^+^ ILC3s at both frequencies and numbers, but decreased RORγt expression at per cell level, which not only caused acquiring ILC1-like gene expression feature in ILC3 cells but also led to generation of a specific CD4^+^NKp46^+^ ILC3 subset. These findings thus characterize CNS9 as an essential regulator controlling the development, stability or plasticity of ILC3s, through fine tuning dose expression of RORγt.

As the master transcription factor of ILC3s, RORγt inhibits ILC1/2-related gene expression. The trans-differentiation from ILC3s to ILC1s, requires down-regulation of RORγt and up-regulation of T-bet expression (18). Previous reports showed that T-bet directly instructed IFN-γ and NKp46 expression (8–10). Therefore, the increased T-bet expression, as a result of decreased RORγt expression, could contribute to the induction of CD4^+^NKp46^+^ ILC3s in CNS9-deficient mice. Consistently, decreased RORγt expression in CNS9-deficient ILC3s resulted in decreased IL-22 and IL-17A secretion and increased IFN-γ production (**Figures 2**, **5A**). Moreover CD4^+^NKp46^+^ ILC3s are likely induced by decreased dose of RORγt expression, as evidenced in both CNS9-deficient and RORγt^GFP^ mice (**Figures 5A–C**). This speculation was further confirmed by the findings that the development of CD4^+^NKp46^+^ ILC3s can be largely prevented by RORγt-overexpression in the bone marrow stage (**Figure 6**). Previously, a small population of CD4^+^NKR^-^LTi cells was reported in adult RORγt^fm^ mice (*Rorc*-Cre^Tg^ heterozygous) (7), and also increased population of NKp46^+^ILC3s observed in RORγt^GFP^ mice compared with WT mice (19), which supports our hypothesis that CNS9 regulates ILC3 subsets development or plasticity through modulating RORγt expression levels.

However, in the mixed bone-marrow reconstituted mice, CNS9 deficiency led to about 75% less frequency of CD4^+^NKp46^+^ ILC3s compared with mice under steady state (**Figures 3B**, **1B**), despite this population was also clearly detected. The difference could be caused by different ages of mice analyzed or altered healthy status in the BM-chimeric mice in the course of experiments. Of note, CNS9-deficient mice had less RORγt expression than RORγt^GFP^ mice, but contained noticeably more abundant CD4^+^NKp46^+^ ILC3s (**Figures 5A–C**, **1B**), further confirmed a dosage-sensitive role of RORγt in control of the development of ILC3s.

Interestingly, we noticed an inverse correlation between *Cd4* and RORγt expression levels in CNS9-deficient ILC3s (**Figures S4A, B**). Considering upregulation of CD4^+^ ILC3s was not found in CNS9-deificent BM-chimeric mice (**Figure 3B**), neither in RORγt^GFP^ mice (**Figure 5C**), suggesting additional mechanism(s) is involved in regulating *Cd4* expression in CNS9-deficient ILC3s, possibly independent on relative dosages of RORγt expression.

In Th17 cells, deletion of CNS9 completely abolishes IL-6 induced RORγt expression and RORγt-directed Th17 cell differentiation, and is thus identified as the dominant *cis*-element in the *Rorc* locus in response to IL-6-STAT3 signaling, with a particularly important role in activating the chromatin structures across the *Rorc* gene locus (12). In contrast to Th17 cells, it has been reported that STAT3 deficiency does not affect RORγt expression in the development of ILC3s (20). Accordingly, CNS9-deficienct mice had normal number and frequency of RORγt^+^ ILC3s compared with WT mice (**Figure 1A**). However, CNS9 deficiency reduced the amounts of RORγt protein in individual ILC3 cells (**Figure 5A**) and altered their composition, inducing a population of CD4^+^NKp46^+^ ILC3s that is not present in normal health mice (**Figure 1B**). These findings thus highlight a distinct role of the same *cis*-acting element between adaptive and innate immune cells.

In this study, defective IL-22 production was observed in mature CNS9-deficient ILC3s stimulated with IL-23 only, but not in the presence of IL-12 and IL-15 (**Figures 2A**, **S2A**). It is known that IL-12 and IL-23 share a common subunit p40, and a common receptor subunit IL-12Rβ1, so the difference is possibly caused by competition or compensation between IL-23 and IL-12 signaling in ILC3s. Interestingly, our scRNA-seq data showed that the mRNA levels of *Il12rb2* (encoding the unique receptor for IL-12) were ~10 times higher in CNS9-deficient than in WT ILC3s (data not shown). Considering the concentration of IL-12 (25 ng/ml) used was 5 fold of IL-23 (5 ng/ml) in *ex vivo* culture, increased IL-12 signaling strength was expected in CNS9-deficient ILC3s, compared to WT ILC3s, which may confer different phenotypes under different stimulation conditions. Since CNS9 functions in a context-dependent manner, whether it regulates fetal or early stage ILC3 development needs further investigation.

In summary, this study, for the first time, investigated the *cis*-regulatory mechanism in ILC3s *via* a genetic approach. Further discovered a distinct essential role of *cis*-element CNS9 in controlling the development or plasticity of ILC3s, through modulating dosing expression of its master transcription factor RORγt. These findings could be useful for understanding the physiological relevance in response to dynamically changing gut-associated microenvironments.

## Data availability statement

The data presented in the study are deposited in the GEO DataSets, accession number is GSE225928.

## Ethics statement

The animal study was reviewed and approved by Ethics Statement All the animal experiments were performed with the use of protocols approved by the Institutional Animal Care and Use Committee of Tsinghua University.

## Author contributions

DC and CD designed the project. DC and HZ performed the experiments and analyzed the data. JG analyzed the bioinformatics data of scRNA-seq. QX helped with the experiments especially overexpress genes in BM cells. XG constructed single cell library. DC, H Z, XW, and CD prepared the manuscript. All authors contributed to the article and approved the submitted version.
