# Supplementary material for: A cis-element at the Rorc locus regulates the development of type 3 innate lymphoid cells
Source: Front Immunol. 2023 Mar 9;14:1105145. doi: 10.3389/fimmu.2023.1105145 (PMC10034404; doi:10.3389/fimmu.2023.1105145)
Supplement: Supplementary file 1 [file DataSheet_1.pdf]

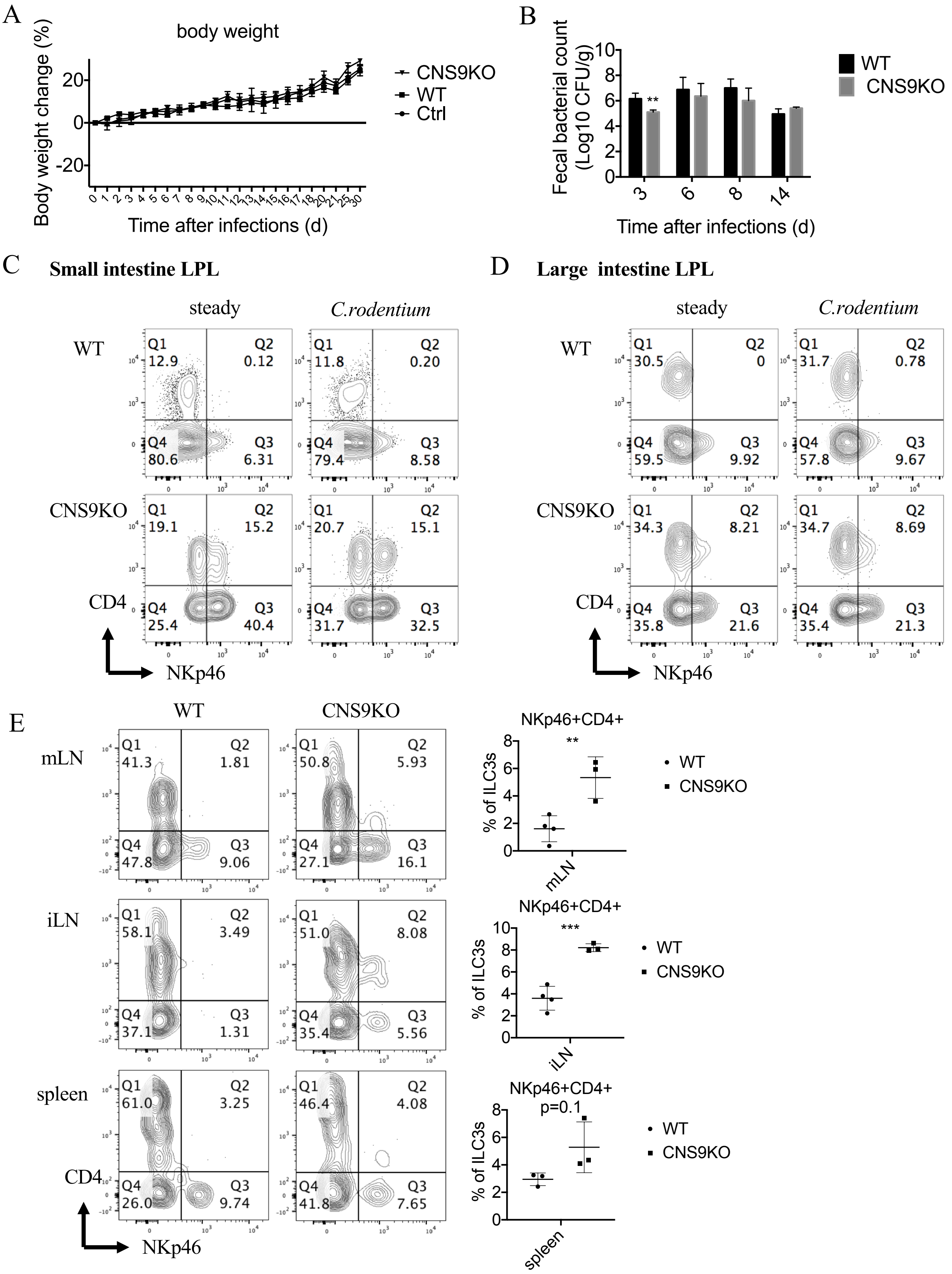

Figure S1. CNS9-deficient mice contained a group of CD4⁺NKp46⁺ ILC3s, related to Figure 1.
(**A-D**) Age- and sex-matched WT and CNS9-deficient mice were inoculated with $2*10^9$ CFU *C.rodentium*.
(**A**) Body weight was monitored until 30 days post infection, and shown as percentage changes (normalized on day 0 data).
(**B**) Fecal bacteria loads were detected on day 3, 6, 8 and 14 post infection, and shown as Log10 CFU/gram of feces (n=5 for WT group and n=3 for CNS9KO group).
ILC3s were isolated from small intestine (**C**) or large intestine (**D**) LPLs of WT and CNS9-deficient mice under steady state and day 8 post *C. rodentium* infections, and analyzed for surface expression of CD4 and NKp46 by flow cytometry.
(**E**) ILC3s were isolated from mesenteric lymphoid node, inguinal lymphoid node and spleen, under steady state of age- and sex-matched WT and CNS9-deficient mice, and analyzed for surface expression of CD4 and NKp46 by flow cytometry. Left: surface staining of CD4 and NKp46 of ILC3s; right: statistic of the staining data.
The data shown are a representative of two independent experiments and presented as mean ± SD.

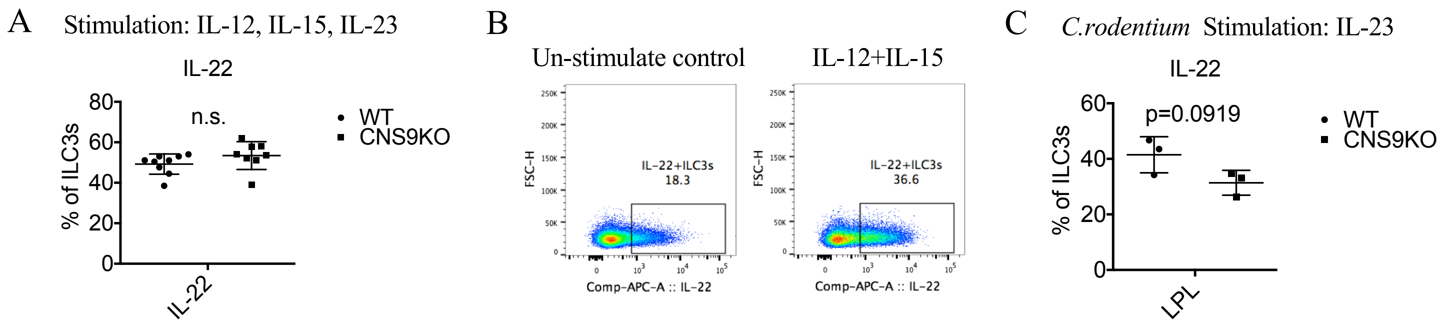

Figure S2. CNS9-deficient ILC3s showed no significant defect of IL-22 production with IL-12&IL-15&IL-23 stimulation, or after *C.rodentium* infection, related to Figure 2.

ILC3s were isolated from the small intestine lamina propria lymphocytes (LPLs) of age- and sex-matched WT and CNS9-deficient mice under steady state. Stimulated *ex vivo* for 4 hours with indicated cytokines before staining with surface markers, RORγt and cytokines, and then analyzed by flow cytometry. The ILC3s were gated as Live CD45+ CD3- CD90+ RORγt cells.

(**A**) Statistics analysis of IL-22 production in ILC3s. The data was performed by combining two independent experiments and presented as mean ± SD.

(**B**) Individual flow cytometry plots showed IL-22 production of WT ILC3s.

(**C**) Age- and sex-matched WT and CNS9-deficient mice were inoculated with $2*10^9$ CFU *C.rodentium*. ILC3s were isolated from small intestine LPLs of WT and CNS9-deficient mice on day 8 post *C.rodentium* infection, and stimulated with IL-23 for 4 hours before analyzing IL-22 production. The ILC3s were gated as Live CD45+ CD3- CD90+ RORγt+ cells. The data are presented as mean ± SD.

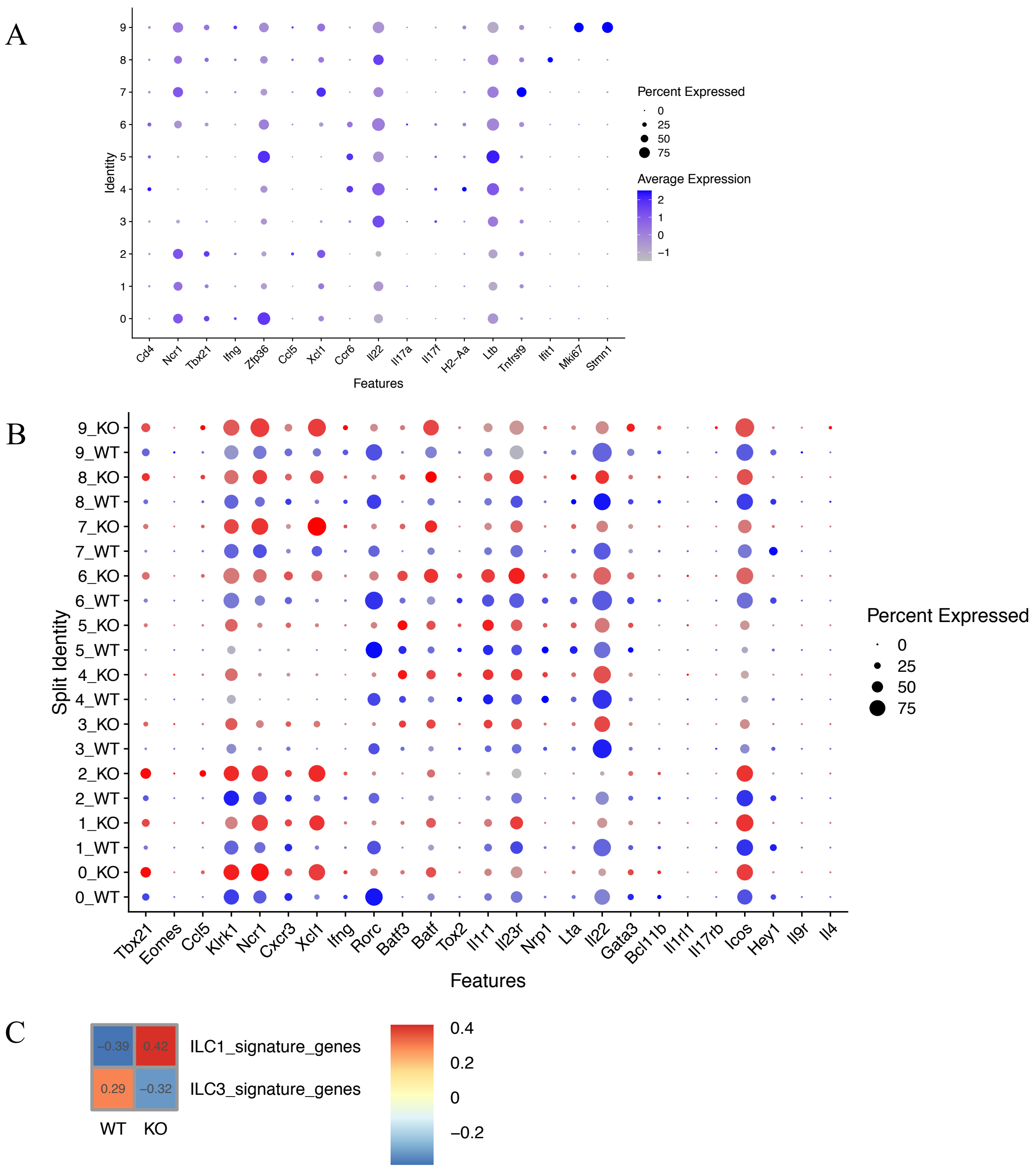

Figure S3. CNS9-deficient ILC3s gained ILC1s signatures, related to Figure 4.

ILC3s were isolated from small intestine LPLs of WT and CNS9-deficient mice by sorting, and processed for single-cell RNA analysis.

(**A**) Expression of selected genes in each cluster.

(**B**) Expression of the ILC1/3/2s signature genes in each cluster between WT and CNS9-deficient ILC3s.

(**C**) ILC1- or ILC3- signature genes (Gury-benari et al., 2016) were analyzed using gene set variation analysis (GSVA), and shown as mean value of total cells.

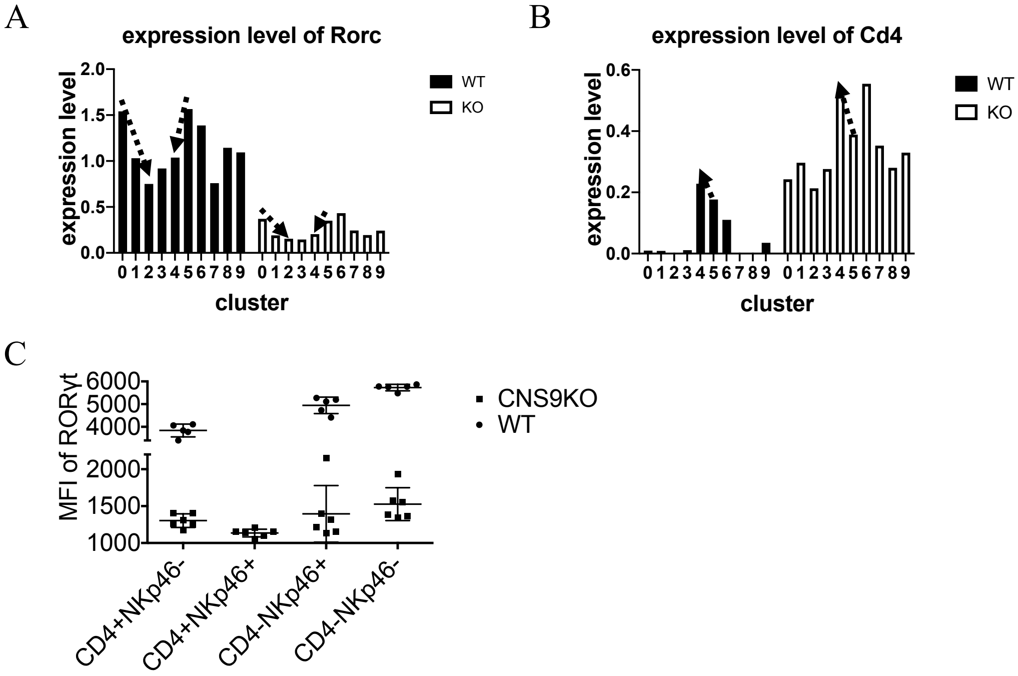

Figure S4. RORγt expression levels in WT and CNS9-deficient ILC3s, related to Figure 5.
(**A-B**) ILC3s were isolated from small intestine LPLs of WT and CNS9-deficient mice by sorting, and processed for single-cell RNA analysis. RNA expression level of *Rorc* (**A**) or *Cd4* (**B**) among each clusters in WT and CNS9-deficient ILC3s.
(**C**) The LPLs were isolated from small intestines of steady state WT and CNS9-deficient mice, stained and analyzed by flow cytometry for RORγt expression among ILC3 subsets. The data shown are a representative of two independent experiments and presented as mean ± SD.

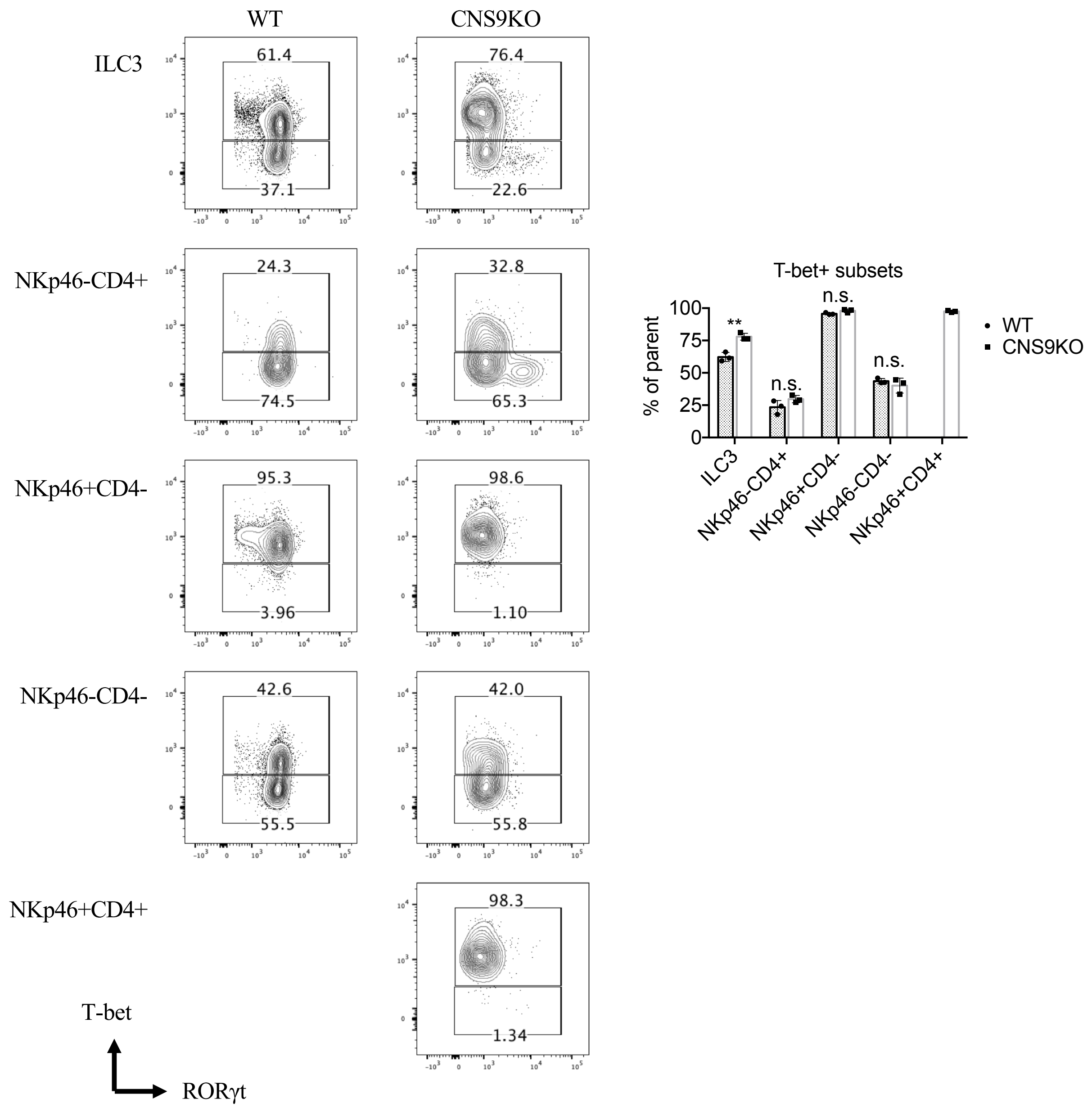

Figure S5. T-bet expression levels in WT and CNS9-deficient ILC3s, related to Figure 5.

The LPLs were isolated from small intestines of steady state WT and CNS9-deficient mice, stained and analyzed by flow cytometry for T-bet and RORγt expression among ILC3 subsets. Representative flow plots (left) and statistic analysis (right) of T-bet+ cells in total ILC3s and different subsets. The data shown are presented as mean ± SD.

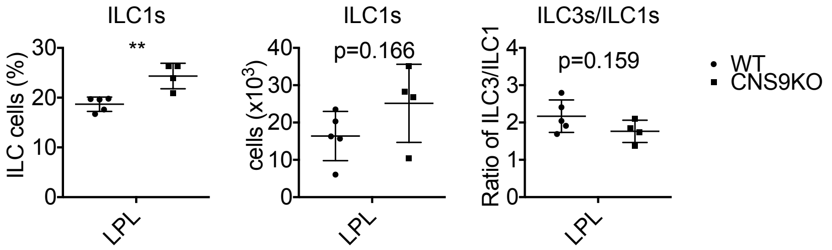

Figure S6. ILC3s didn't transit to ILC1s after CNS9 deficiency, related to Figure 6.
The LPLs were isolated from small intestines of steady state WT and CNS9-deficient mice, stained and analyzed by flow cytometry, in which ILC3s were gated as Live $CD45^+$ $CD3^-$ $CD90^+$ $ROR\gamma t^+$ cells, and ILC1s were gated as Live $CD45^+$ $CD3^-$ $CD90^+$ $ROR\gamma t^-$ $NKp46^+$ cells. The frequencies and cell numbers of ILC1s, and the ratios of ILC3s *versus* ILC1s in WT and CNS9-deficient mice.
The data shown are a representative of two independent experiments and presented as mean ± SD.
